# How to know who you are through your short video selfies?—Capturing personality *via* short video selfies

**DOI:** 10.3389/fpsyg.2023.1072344

**Published:** 2023-03-06

**Authors:** Zhiwen Dong, Tian Xie

**Affiliations:** ^1^Department of Psychology, Philosophy School, Wuhan University, Wuhan, China; ^2^School of Psychological and Cognitive Sciences and Beijing Key Laboratory of Behavior and Mental Health, Peking University, Beijing, China

**Keywords:** big five personality, short video selfie, TikTok (Douyin in China), the lens model, zero-acquaintance judgment

## Abstract

The extant literature has accumulated enormous knowledge on personality prediction from digital records on social networking sites (e.g., photo selfies). However, little is known about how short video selfies reflect their owner’s personality and how people judge others’ personalities from short video selfies. Taking short video selfies is very popular today; many people are willing to share their short video selfies with others. Based on the lens model theory, it is expected that one’s personality is associated with short video selfies. By analyzing 177 Chinese TikTok (Douyin in China) users’ short video selfies and their Big Five personalities, it showed that specific cues in short video selfies related to agreeableness, conscientiousness, neuroticism, and openness. But only extraversion could be predicted by short video selfies accurately. This study is the first to reveal personality-related cues in short video selfies and has practical implications for both short video platforms and their users.

## Introduction

Personality could be captured from not only individuals’ environments (e.g., books or magazines in one’s bedroom, Gosling et al., 2002), but also digital records on social networking sites (Azucar et al., 2018). Research has shown that personality could be predicted through a tiny movement (e.g., clicking “Like,” commenting) in social media and a considerable body of knowledge has accrued from research on personality expression and perception in digital records (e.g., Kosinski et al., 2013; Qiu et al., 2015). For example, Kosinski et al. (2013) collected “Like” data from Facebook and found that openness was strongly associated with “Like.” Moreover, prior studies demonstrated that personality expression in social media differs in different situations (Gosling et al., 2002). For instance, Conscientiousness was related to friendliness in Facebook profiles (Hall and Pennington, 2013). Openness was associated with sociability in Instagram profiles (Cooper et al., 2020). Extraverts are active in using different functions to improve the quality of short videos in TikTok (Meng and Leung, 2021). Even so, little is known about how personality is related to short video selfies.

Short video selfies refer to short self-portrait videos taken by oneself using a smartphone for posting on short video platforms. The short video apps provide people with the convenience of taking short video selfies and sharing them with the public. Since its launch in 2016, the leading short video platform TikTok has gained global success (Scherr and Wang, 2021). In September 2021, the number of monthly active TikTok users reached 1 billion globally, representing 45% growth compared to monthly active users in July 2020 (Statista, 2022). Compared to other social networking sites (i.e., Sina Weibo), people are more proactive in posting short video selfies on short video platforms (CINIC, 2021). Furthermore, compared with pictures, sounds, and texts, short video selfies contain richer cues that could reflect an individual’s personality. For example, the movement could only be seen in videos. In this line, we might capture people’s personalities from their short video selfies.

The study aims to identify personality-related cues in short video selfies and examine how people predict personality based on short video selfies. Since video is like the combination of many continuous photos and sounds, the studies on personality and photos, selfies, and videos, and the lens model (the theoretical basis of the current study) are reviewed below. Moreover, people in different levels of demographic variables (i.e., gender and age) might differentially use social applications (e.g., Qiu et al., 2015; Sorokowska et al., 2016; Kim and Chock, 2017; Arpaci, 2018; Kaurin et al., 2018) and prior studies have found that the stereotypes of gender and age could influence observers’ personality judgment (Kenny et al., 1992). Thus, the present study will examine the relationship between demographic variables (i.e., gender and age) and short video selfies cues.

## Background research

### Personality expression in photos, selfies, and videos

Research has shown a connection between self-reported personality and photos based on different photographs, such as standard photographs and spontaneous photographs (e.g., Naumann et al., 2009; Nestler et al., 2012; Cooper et al., 2020; Campbell et al., 2022). For example, Naumann et al. (2009) coded the participants’ spontaneous photographs and found that extraversion was positively associated with smiling. Nestler et al. (2012) found that conscientiousness was positively associated with attractiveness, and openness was positively related to the volume of mouth based on the standard photographs. Other studies used photos posted on social media taken by experimenters. They found conscientiousness was positively associated with self-generated albums and video uploads on Facebook, while neuroticism and extraversion were positively associated with photo uploads on Facebook (Eftekhar et al., 2014). Moreover, Cooper et al. (2020) accessed one’s personality through the situational and behavioral features of photos on Instagram, and found that openness was positively related to sociability.

Prior research has provided robust evidence on the relationship between selfies posted on social media and the Big Five personalities (Qiu et al., 2015; Sorokowska et al., 2016; Choi et al., 2017). Several studies investigated the question through self-reported data. For example, Sorokowska et al. (2016) analyzed the frequency of online selfie-posting on various online social networking, demonstrating that extroverts prefer to post selfies on social media. Choi et al. (2017) committed that openness was associated with online social connections negatively using selfies. Other studies explored the relationship by coding the selfies posted on social media. For example, Qiu et al. (2015) coded users’ selfies on Sina Weibo and found their committed agreeableness was positively associated with eyes looking at the camera in selfies. Kaurin et al. (2018) coded the recent selfies uploaded on social media and found that extraversion was positively associated with selfies with a pet. In addition, research has shown a connection between narcissistic personality and selfies (e.g., Shane-Simpson et al., 2020; Wang et al., 2020; Koterba et al., 2021). For example, Wang et al. (2020) found that narcissistic individuals are more likely to post their selfies on social media because they are satisfied with their bodies.

Moreover, prior research has tried to explore the relationship between videos and personality. For example, Potard et al. (2020) investigated the relationship between video game players’ profiles and Big Five personalities, and found that role-playing video games were positively associated with openness. Hickman et al. (2022) explored the interviewers’ Big Five personalities’ assessment through automated video interviews (AVIs), and found that AVI personality assessments had evidence of reliability. Kaurin et al. (2018) coded the self-introductory videos and found the highest correlation between personality and videos. However, they did not focus on short video selfies in non-laboratory conditions (e.g., the selfies in TikTok). Furthermore, researchers have examined the relationship between Big Five personality and short video (i.e., TikTok) engagement behaviors, and found that people with high extraversion are active in using different functions to improve the quality of short videos (Meng and Leung, 2021).

Compared to photos and selfies, short video selfies could provide unique cues that may reflect people’s personalities. One of the motivations for shooting and submitting short videos is recording and sharing (Dong and Xie, 2019). As the motivation for recording their owners’ lives, short video selfies may contain more vital cues of personality, which could provide a better view of personality than photos and selfies. Short video selfies give individuals enough time to perform themselves rather than a flash recording like photos. Specifically, users could present their activities in the short videos, which contain more information such as their emotions and voices. Moreover, compared to photos and selfies, watchers could receive the information more straightly and could better understand what and why uploaders want to send messages to their audiences. Thus, short video selfies may contain richer cues unavailable in photos and selfies.

### The effect of age and gender on personality expression on social networking

Prior research suggested that demographic variables (i.e., gender and age) of uploaders could influence the relationship between personality expression and selfies on social networking sites (e.g., Qiu et al., 2015; Sorokowska et al., 2016; Kim and Chock, 2017; Arpaci, 2018; Kaurin et al., 2018). On the one hand, it is different to use social applications among people in various levels of demographic variables (i.e., gender and age). For instance, compared to men, women are likely to spend more time on selfies-post, and the connection of narcissism with selfie-posting behavior is significant for men rather than women (Arpaci, 2018). Kim and Chock (2017) suggested that the need for popularity promotes solo selfies among men, not women. Furthermore, they found that younger people are more likely to post solo selfies than older people (Weiser, 2015). On the other hand, observers’ personality judgment could be influenced by the stereotypes of gender and age (Kenny et al., 1992). For example, Qiu et al. (2012) explored how words on Twitter reflect users’ personalities and found that observers might rely on the stereotypes of gender and age rather than valid linguistic cues when judging personality.

According to a survey of TikTok (Douyin in China) in 2018 (iResearch, 2020), TikTok users were more likely to be females and younger. Over 50% users of TikTok are females (in 2017 and 2018, female accounted for 63.0 and 59.0%, respectively). TikTok users consist of people from children to older, and most users are 21–25 years (in 2018, males and females accounted for 40.1 and 50.0%, respectively). Thus, the age and gender of TikTok uploaders might influence the relationship between personality expression and short video selfies.

### The lens model

**Figure 1:**
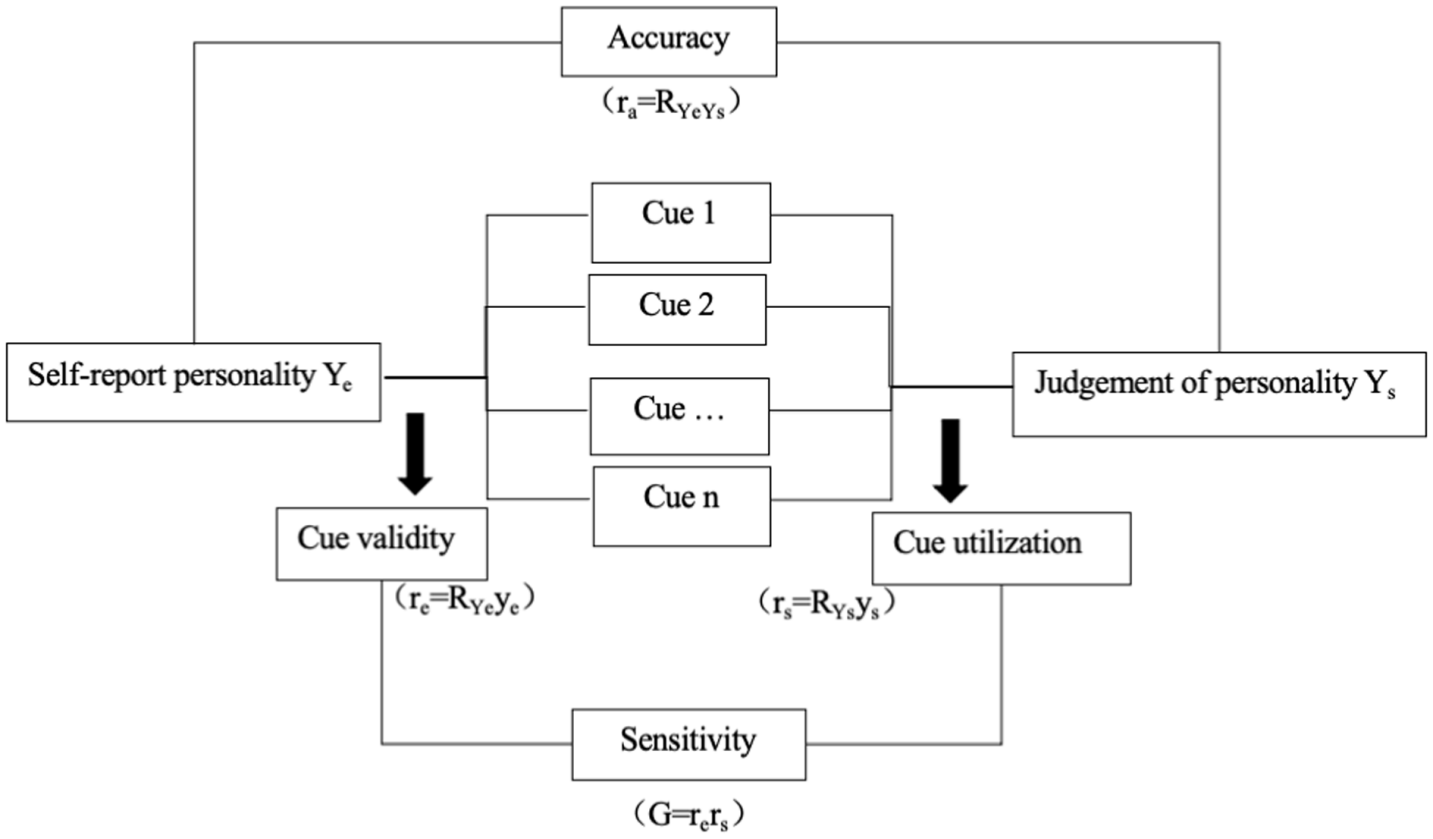
Diagram of the lens model. Adapted from “Determinants of Linear Judgment: A Meta-Analysis of Lens-Model Studies,” by Karelia and Hogarth (2008), and from “An Integrative Lens Model Approach to Bias and Accuracy in Human Inferences: Hindsight Effects and Knowledge Updating in Personality Judgments,” by Nestler et al. (2012), respectively, by the American Psychological Association.

The lens model helped define and learn interpersonal judgment (Brunswik, 1956) and was widely used in personality judgment (e.g., Nestler et al., 2012; Qiu et al., 2012, 2015; Kaurin et al., 2018; Proyer and Brauer, 2018). The lens model hypothesizes that several visual cues could characterize a given personality trait. And observers could recognize the given trait through the same cues (Qiu et al., 2015). For example, personality traits such as neuroticism could be explained by unstable mood (observable cue). Meanwhile, people could judge neuroticism based on varying perspectives. In other words, the accuracy of personality judgment from the lens model depends on the observable cues from the environment. The cues could be considered as a mediator between self-report personality and assessment of personality (Qiu et al., 2015). Based on the hypothesis, four indicators will be used to reflect the accuracy and validation of short video cues in the judgment of personality (see Figure 1).

Accuracy (r_a_) connected the self-report personality with the personality judgment, with a stronger correlation indicating more accuracy. Cue validity (r_e_) is the degree of association between cues (y_e_) and self-report personality (Y_e_), with a stronger correlation suggesting better validity. Cue utilization (r_s_) is the link between cues (y_s_) and the judgment of personality (Y_s_), and a strong correlation indicates that the cue is more utilized when forming personality judgments. Cue sensitivity (G) is the correlation between cue validity (r_e_) and cue utilization (r_s_), with a higher correlation indicating that cues could reflect the given personality well. We will adopt this model to examine how personality is expressed in short videos and what given cues people could use to judge one’s personality from the short videos.

### The present study

The present study aims to predict one’s personality through short video selfies. Based on the lens model, the study firstly detected valid cues in short video selfies associated with self-reported personality, then identified the potential cues observers may rely on to make personality judgments, and finally examined the impact of gender and age on observers’ personality judgment. The short video selfies from TikTok were collected and rated to identify the personality and selfie cues in the short videos.

## Method

### Participants

Participants were collected through an online survey platform.^1^ We selected 2,092 Chinese TikTok (Douyin in China) users who recorded and posted short videos and sent each user a participation request, and 400 users participated in return. We deleted 205 invalid participants (including advertising business, the number of videos less than 10) and deleted 18 participants who only made private videos. A total of 177 users participated in the current study (61 men, 116 women, *M_age_* = 26.78, *S.D.* = 6.27, the average number of short videos = 112.32).

### Procedure

All participants completed a two-part online survey, were informed about the study, and provided informed written consent. The first part comprises the Big Five personality scale (Mini-IPIP, Donnellan et al., 2006). The second part asked participants about their TikTok user ID, the frequency of recording videos and posting them online, and demographic variables (i.e., gender and age).

Then, we observed the online videos of all participants and identified which of these videos were selfies (0 = No, 1 = Yes). To code the short video selfies, we first selected photos/selfies-coding that are appropriate for coding short video selfies from past researches, because short video selfies were similar to photo selfies (e.g., Qiu et al., 2015; Kaurin et al., 2018). Combining the characteristics of short video selfies, the codes were retained as follows: Camera in front (*0 = face away from the camera, 1 = face the camera*); Eyes looking at the camera (*0 = not looking at the camera, 1 = looking at the camera*); Emotional unchanged (*0 = emotional changed, 1 = emotional unchanged*); Smile (*0 = not smile, 1 = smile*); Face visibility (*0 = part of face, 1 = complete face*); The whole body(*0 = part of body, 1 = whole body*); Camera height (*0 = below or the same level of head, 1 = above head*); Photoshop editing (*0 = no photoshop editing, 1 = photoshop editing*); and Alone (*0 = over 1 person in the short video selfies, 1 = only 1 person in the short video selfies*). Moreover, we added cues that are unique for short video selfies. First, users can choose their performances or real lives to share with others through the short video platform. Thus, we added the code of “acting” (*0 = real life, 1 = performance/acting*). Second, people could control the shooting time and switch camera angles freely when taking short video selfies. Thus, the code of “shooting from the same angle” (*0 = shooting from the different angles, 1 = shooting from the same angle*) was used in the study. Third, similar to photoshop editing, users could edit their short video selfies. For example, users could add some special effects that the apps provided freely and present subtitles to embellish their short video selfies. Therefore, the codes of “video effects” (e.g., *switch scene; 0 = no video effects, 1 = video effects*) and “subtitle” (*0 = no subtitle, 1 = subtitle*) were added to the cues. Forth, users could take short video selfies with the help of their surroundings and tools. Thus, the codes of “situational cues” (e.g., *classroom*; *0 = no situational cues*, *1 = situational cues*) and “tools” (e.g., *besom*; *0 = no tools*, *1 = tools*) were used. Finally, short video selfies contain not only selfies, but also background music. Users could add a period of background music to their short videos freely or choose the original sound as background for short videos. According to interactionist theories of music, the musical environments that people select could reflect their psychological traits (Buss, 1987; Anderson et al., 2021). Therefore, the background music that users selected in the short video selfies (*0 = no background music*, *1 = background music*) was adopted as a cue to capture users’ personalities. Consequently, the study contained a total of 17 cues: Selfie; Acting; Camera in front; Shooting from the same angle; Eyes looking at the camera; Emotional unchanged; Smile; Face visibility; The whole body; Camera height; Video effects; Photoshop editing; Subtitle; Background music; Situational cues; Tools; and Alone.

Two independent raters were chosen to code the cues. We adopted zero-acquaintance personality judgment following prior studies (e.g., Borkenau and Liebler, 1992; Krämer and Winter, 2008; Qiu et al., 2015), which means all coders are unfamiliar with participants. The coders need to observe all short videos of participants, code them, and then judge whether the short videos match the above cues. Moreover, the coding consistency should be at least 90%. If an item received inconsistent coding from two raters, another rater recoded the item and made the final judgment. Then, the number of cues was obtained and the proportion of cues was calculated (e.g., the proportion of acting = the number of acting/the number of selfies *100%). Finally, two undergraduate students who majored in psychology and were unfamiliar with participants, were selected as observer1 and observer2. They browsed each short video and rated their impression of the selfie owner’s personality using the same Big Five personality scale that the participants used. Then the observers’ ratings were aggregated by calculating the average score of observed personalities.

### Measures

#### Personality

Personality was measured by the Big Five personality scale (Mini-IPIP, Donnellan et al., 2006). The scale consisted 20 items (e.g., “friendliness”) and five dimensions: extraversion (Cronbach’s *α* = 0.60), openness (Cronbach’s *α* = 0.61), neuroticism (Cronbach’s *α* = 0.66), agreeableness (Cronbach’s *α* = 0.65), and conscientiousness (Cronbach’s *α* = 0.34). The participants rated each item on a five-point scale ranging from 1 = strongly disagree to 5 = strongly agree.

## Results

### Accuracy

As shown in Table 1, intra-class correlations (ICC) of observers were calculated to measure judgment consensus of selfie owners’ personality traits (Vazire and Mehl, 2008). The results showed that observers’ ratings reached a moderate consensus on all five personality dimensions, which means that observers might use the same cues for rating short video owners’ personalities. Specifically, the consensus of observers’ rating of openness is the highest (*ICC* = 0.40). On the other hand, we used aggregated observer accuracy (i.e., the correlation between the aggregated observers’ rating and self-report personality) to measure the accuracy of observers’ personality judgment. However, the reliability of aggregated observers’ ratings on personality in a single analysis (i.e., aggregated accuracy) might be boosted because of aggregation (Vazire and Mehl, 2008). Thus, to correct the bias, we chose the single observer (observer1 and observer2) accuracy (i.e., the correlations between the single observer’s rating and self-report personality). As shown in Table 1, the results showed that the correlation between self-report and aggregated observers’ rating on extraversion (*r* = 0.16, *p* < 0.05), the correlation between self-report and observer1’s rating on extraversion (*r* = 0.18, *p* < 0.05), and the correlation between self-report and observer2’s rating on extraversion (*r* = 0.17, *p* < 0.05) are significant, which means that observers could accurately predict extraversion based on short video selfie cues. And we found that the other four dimensions (i.e., openness, agreeableness, neuroticism, and conscientiousness) did not obtain significant correlations.

**Table 1:** Self and observer rating of personality: consensus, accuracy, and vector correlation.

	Self-rating		*Concensus*	Accuracy			Vector correlation
	*M*	*SD*	*ICC*	Aggregate	Observer1	Observer2	
Extraversion	3.10	0.68	0.33^**^	0.16^*^	0.18^*^	0.17^*^	0.70^**^
Agreeableness	3.60	0.60	0.32^**^	0.03	−0.02	0.07	0.10
Conscientiousness	3.43	0.61	0.38^**^	0.07	−0.05	0.08	0.10
Neuroticism	3.13	0.72	0.34^**^	−0.06	−0.10	0.01	−0.01
Openness	3.40	0.44	0.40^***^	−0.03	−0.04	−0.01	0.14

*N* = 177. The intraclass correlation (ICC) was used to measure the consensus of the rating of personality among observers. Aggregated observer accuracy: the correlation between the aggregated observers’ rating and self-report personality. Observer1 and Observer2 accuracy: the correlations between observer1 and observer2’s rating and self-report personality. Vector correlation: the correlation between cue utilization and cue validity after Fisher’s r-to-Z transformation. ^*^p < 0.05; ^**^p < 0.01; ^***^p < 0.001.

### Sensitivity

Following prior research (Funder and Sneed, 1993; Back et al., 2010; Qiu et al., 2012, 2015), to match the pattern of cue utilization and cue validity, we performed vector correlations to test the cue sensitivity of observers toward valid short video selfies cues. The correlations between cue utilization and cue validity were calculated after Fisher’s r-to-Z transformation, and a strong correlation indicates that observers used valid cues to generate accurate judgment of personality. As shown in Table 1, we found a strong vector correlation for extraversion (*r* = 0.70, *p* < 0.01), indicating that observers used valid cues to generate accurate judgment of extraversion. The other four dimensions (i.e., openness, agreeableness, neuroticism, and conscientiousness) did not obtain significant vector correlations.

### Cue validity

Cue validity was assessed through the correlation between participants’ self-report personality and cues in short video selfies after controlling age and gender (see Table 2). The proportion of cues in short video selfies was used to analyze the cue validity. Extraversion, agreeableness, consciousness, and openness were correlated with selfies, respectively (*r* = 0.22, 0.26, 0.16, 0.17, *p* < 0.05), while neuroticism was not related to selfies (*r* = 0.06, *p* > 0.05), indicating that people with high extraversion, agreeableness consciousness, and openness are likely to post their short video selfies on TikTok. Extraversion, consciousness, and openness were positively associated with acting (*r* = 0.18, 0.14, 0.17, *p* < 0.05), suggesting that people with high extraversion, consciousness, and openness are likely to perform on short video platforms. Extraversion was related to situational cues (*r* = 0.17, *p* < 0.05), suggesting that people in high extroversion are shooting selfies in special situations, such as working situations. Agreeableness was positively connected with the smile (*r* = 0.18, *p* < 0.05), which is consistent with the prior research (Seidman, 2013; Qiu et al., 2015). Neuroticism was negatively associated with video effects and photoshop editing (*r* = −0.13, −0.20, *p* < 0.05), suggesting neurotic individuals tend to upload original selfies to short video platforms. Openness was negatively related to shooting from the same angle and tools (*r* = −0.15, −0.16, *p* < 0.05), while positively associated with camera height (*r* = 0.20, *p* < 0.05), indicating that people with high openness are likely to shoot videos in different angles and above head, and less to use tools in their videos.

**Table 2:** The lens model analysis: Cue-validity (Cue-utilization) correlation.

Cue validity							Short video selfies cues	Cue utilization				
Extra.	Agree.	Cons.	Neur.	Open.	Gender	Age		Extra.	Agree.	Cons.	Neur.	Open.
0.22^**^	0.26^**^	0.16^*^	0.06	0.17^*^	0.20^**^	−0.04	Selfies	0.30^**^	0.03	0.04	−0.10	0.21^**^
0.18^**^	0.12	0.14^*^	−0.10	0.17^*^	−0.05	0.04	Acting	0.22^**^	0.08	−0.01	−0.14^*^	0.12
0.02	0.02	−0.03	0.06	0.01	0.21^**^	−0.01	Camera in front	−0.08	−0.07	0.06	0.14^*^	−0.05
−0.09	−0.12	−0.04	0.01	−0.15^*^	0.16^*^	−0.08	Shooting from the same angle	−0.06	0.01	−0.05	−0.06	0.06
0.00	0.04	−0.10	0.01	−0.02	0.37^**^	0.05	Eyes looking at the camera	0.00	−0.10	0.01	0.05	0.09
−0.02	0.05	−0.02	−0.02	−0.04	0.07	0.03	Emotion unchanged	0.06	−0.03	0.09	0.00	0.06
0.10	0.18^*^	0.09	−0.04	−0.02	0.32^**^	0.04	Smile	−0.04	0.14^*^	0.08	−0.01	0.07
0.13	0.08	0.05	−0.05	0.10	0.11	0.02	Face visibility	0.05	−0.16^*^	−0.06	0.06	0.05
−0.03	0.01	0.02	−0.02	−0.07	−0.31^**^	0.07	The whole body in video	0.11	0.06	0.00	−0.16^*^	0.05
0.01	0.14^*^	0.09	−0.01	0.20^**^	−0.09	−0.05	Camera height	0.08	−0.10	0.03	0.01	0.08
0.01	0.13^*^	−0.01	−0.13^*^	−0.07	0.31^**^	−0.17^*^	Video effects	−0.02	0.03	−0.14^*^	0.07	−0.02
0.03	0.12	0.08	−0.20^**^	0.01	0.39^**^	−0.04	Photoshop editing	0.18^*^	0.14^*^	−0.10	0.18^*^	0.11
0.04	0.00	−0.12	−0.08	0.11	0.06	−0.01	Subtitle	0.03	0.09	0.16^*^	−0.01	0.15^*^
0.12	0.13^*^	0.06	−0.11	0.07	−0.15^*^	−0.07	Background music	0.04	0.04	0.02	−0.10	−0.03
0.17^*^	0.07	0.14^*^	−0.09	0.03	−0.05	0.07	Situational cues	0.05	0.10	0.05	−0.08	−0.06
−0.09	−0.09	−0.12	0.05	−0.16^*^	−0.13	−0.04	Tools	−0.14^*^	0.03	−0.07	0.03	−0.07
−0.05	0.01	−0.06	−0.01	−0.05	0.09	−0.09	Alone	−0.04	−0.05	−0.03	0.01	−0.07

*N* = 177. ^*^p < 0.05; ^**^p < 0.01. Gender: 1 = Male, 2 = Female.

### Cue utilization

Cue utilization was assessed by connecting short video selfie cues to observers’ ratings of personality after controlling age and gender (See Table 2). Ratings of extraversion were positively associated with selfies, acting, and photoshop editing (*r* = 0.30, 0.22, 0.18, *p* < 0.05), which is consistent with the characteristics of extraversion. People with high extroversion tend to be more sociable and high-performance (Amichai-Hamburger and Vinitzky, 2010). The results suggested that observers considered that people with high extroversion upload selfies and acting shows to short video platforms to convey their social contact and performance. However, ratings of extraversion were negatively associated with tools (*r* = −0.14, *p* < 0.05), suggesting that people with high extraversion are less likely to use tools in short videos. Agreeableness ratings were positively related to smile and photoshop editing (*r* = 0.14, 0.14, *p* < 0.05) and negatively related to face visibility (*r* = −0.16, *p* < 0.05), indicating that observers considered that agreeable individuals might hide their part of the face to present their value of social affiliation (Marshall et al., 2015). Ratings of conscientiousness were negatively related to video effects (*r* = −0.14, *p* < 0.05), suggesting that conscientious individuals are likely to convey their authentic selves to others. And ratings of conscientiousness were positively connected to subtitles (*r* = 0.16, *p* < 0.05), indicating that observers considered that conscientious individuals do more work in their video to convey their hardworking (Costa and McCrae, 1992). Neuroticism ratings were positively associated with the camera in front and photoshop editing (*r* = 0.14, 0.18, *p* < 0.05) while negatively associated with acting and the whole body in the video (*r* = −0.14, −0.16, *p* < 0.05), suggesting that neurotic individuals tend to concern their impression management edit their short videos and upload their selfies to present an ideal self (Marshall et al., 2015; Bowden-Green et al., 2021). Openness ratings were positively associated with selfies (*r* = 0.21, *p* < 0.01) and subtitles (*r* = 0.15, *p* < 0.05), suggesting that people with high openness are likely to post their short video selfies on social media and add subtitles in short video selfies.

### The mediating role of short video selfie cues in self-other report personality

Based on the correlation results, we tested the mediating role of the short video selfie cues in self-other report agreeableness and extraversion using SPSS (Hayes, 2022; Model 4), because the smile is the only cue related to both observer and self-rating agreeableness, and acting is the only cue correlated with both observer and self-rating extraversion. First, the prior study suggested that agreeableness was positively associated with smiling and positive emotion words (Mehl et al., 2006; Qiu et al., 2015). In the study, smile was positively related to self-report agreeableness and other-rating agreeableness. However, in the mediating analysis, controlling for gender and age, the indirect effect was not significant (*indirect effect size* = 0.02, *SE* = 0.02, 95%*CI* = [−0.002, 0.06]). Second, the results showed that acting was positively related to self-reported and other-rating extraversion. In the present study, controlling for gender and age, the total effect of self-report extraversion on other-rating extraversion was significant (*effect size* = 0.15, *SE* = 0.07, 95%*CI* = [0.01, 0.29]), the direct effect of self-report extraversion on other-rating extraversion was significant (*effect size* = 0.12, *SE* = 0.07, 95%*CI* = [−0.02, 0.26]), and the indirect effect of self-report extraversion on other-rating extraversion through acting was significant (*effect size* = 0.03, *SE* = 0.02, 95%*CI* = [0.002, 0.08]). The result suggested that the acting partly mediated the self-other report extraversion. The pattern of results was identical without controlling for gender and age. Specifically, without controlling for gender and age, the indirect effect of self-report extraversion on other-rating extraversion through acting was still significant (*effect size* = 0.03, *SE* = 0.02, 95%*CI* = [0.001, 0.08]).

### Gender and age effect

The correlation between short video selfies cues and gender and age are present in Table 2. Gender was positively correlated with selfies (*r* = 0.20, *p* < 0.01), suggesting that compared to men, women are more likely to post their short video selfies on social media, which is consistent with prior studies (Sorokowska et al., 2016). Regarding the relation between selfie cues and personality, gender was positively associated with the camera in front (*r* = 0.21, *p* < 0.01), shooting from the same angle (*r* = 0.16, *p* < 0.05), eyes looking at the camera (*r* = 0.37, *p* < 0.01), smile (*r* = 0.32, *p* < 0.01), video effects (*r* = 0.31, *p* < 0.01), and photoshop editing (*r* = 0.39, *p* < 0.01), suggesting that compared to men, women are more likely to shoot short videos from the one angle, smile in the videos, and keep looking at the camera in front, which is consistent with prior study (Qiu et al., 2015; Arpaci, 2018). Furthermore, they prefer to edit the short video selfies through tools provided by short video platforms, which is consistent with prior studies (Qiu et al., 2015). Moreover, gender was negatively associated with the whole body in the video (*r* = −0.31, *p* < 0.01) and background music (*r* = −0.15, *p* < 0.05), suggesting that compared to men, women are more likely to present a part of the body such as head and less likely to use the background music in short video selfies. Regarding the relation between short video selfies cues and age, the results showed that age was negatively correlated with video effects (*r* = −0.17, *p* < 0.05), suggesting that older are less likely to use video effects to modify their videos and present original images to others.

Moreover, prior studies have evidenced that there are stereotypes in observer judgments of personality (e.g., Kenny et al., 1992; Gosling et al., 2002; Graham and Gosling, 2012; Qiu et al., 2015). Thus, the influence of stereotypes of age and gender on observers’ personality judgment was examined. Following prior studies (e.g., Kenny et al., 1992; Gosling et al., 2002; Qiu et al., 2015), we tested cue utilization without controlling age and gender. Compared to the cue utilization controlling age and gender, three of the initial 14 significant correlations became insignificant [i.e., the correlation between acting and neuroticism (*r* = −0.10, *p* > 0.05), camera in front and neuroticism (*r* = 0.06, *p* > 0.05), tools, and extraversion (*r* = −0.07, *p* > 0.05)] became insignificant, and 11 (78.5%) out of 14 correlations remained statistically significant, without controlling age and gender, suggesting that observers’ personality judgment mainly relied on short video selfie cues rather than the stereotypes of gender and age.

## Discussion

The current study explored the relationship between personality and social media use by testing personality expression and perception in short video selfies, a new form of self-portrait in social media. Through the lens model, a few short video selfie cues were identified in personality expression and perception. For example, extroverts prefer to post selfies and act on short video platforms, and people with high agreeableness prefer smiling in short video selfies. Specifically, acting mediated the self-report extraversion and other-rating extraversion. Moreover, regarding accuracy and sensitivity, the study found similar results to previous studies on photos and selfies (Naumann et al., 2009; Qiu et al., 2015). In short video selfies, raters were sensitive to extraversion when rating individual personality traits based on coding cues, which is different from prior findings where raters were most susceptible to openness in photos and selfies (Qiu et al., 2015). Finally, the relationship between demographic variables (i.e., gender and age) and short video selfies was tested, and the impact of the stereotypes of age and gender on personality judgment was excluded.

### Implications

The findings of this study have important theoretical implications. First, the results contribute to the literature on personality expression on social networking sites. The study reveals that short video selfies contain valid personality-related cues, expanding prior findings that people’s digital footprints on social media could reveal their personality traits (Amichai-Hamburger and Vinitzky, 2010; Barry et al., 2019; Qiu et al., 2019). Moreover, the relationship between personality expression and perception in photos and selfies on social media was revealed through the lens model (Naumann et al., 2009; Ong et al., 2011; Nestler et al., 2012; Qiu et al., 2015). Thus, the findings of short video selfies increase our understanding of personality expression and perception on social media through the lens model. Second, the study expands the scope of research on personality and short video selfies by showing that personality might be related to short video selfie cues in non-laboratory conditions. Prior studies identified the relationship between personality and standardized videos in the lab condition (e.g., Kaurin et al., 2018); however, based on the present study, more short video cues were identified, such as acting and background music.

Our research has several vital practical implications. On the one hand, with the growing number of short video selfies, it is meaningful to understand how short video selfies reflect personality. Prior studies have demonstrated that personality could be an important antecedent of job performance (Judge et al., 1999), health (Soldz and Vaillant, 1999), and subjective well-being (Hayes and Joseph, 2003). Short video selfies could represent a fast and affordable method to predict personality, which could reveal some health-related information (Gale et al., 2015; Azucar et al., 2018) and improve the efficacy of interventions (Chapman et al., 2014). On the other hand, a few commercial applications (e.g., TikTok and Kuaishou) could improve their recommender system by learning personality and enhancing user experience (Farnadi et al., 2016).

### Limitations and future direction

The present study has several limitations which need to be addressed in the future. First, we recruited users only from TikTok. Future research needs to examine whether our findings can be generalized to other short video platforms such as Kuaishou and WeSee. Second, prior research has examined the relationship between personality with the different elements of music, such as music genres, acoustic features, psychological attributes, and lyrics (Rentfrow and Gosling, 2003; Rentfrow et al., 2011; Greenberg et al., 2016; Pilgrim et al., 2017; Qiu et al., 2019). However, short videos are not classified. Future research could investigate the latent factors that express short videos by categorizing short videos and then explore the relationship between the latent factors and personality. Third, prior studies suggested the relationship between personality expression and music preference (Rentfrow and Gosling, 2003; Qiu et al., 2019). Nevertheless, the short video cues encoded in the study were limited to the presence or absence of background music. Future research could explore the connection of personality expression with music preference in short videos. Forth, it is far from enough that 17 short video selfies cues were explored in the study, other important cues (i.e., the shooting position change) could be investigated in future studies. Fifth, in the study, only two coders participated in the work of judgment of personality, which might influence the accuracy of results. Future studies could invite more participants to judge the personality of short video users as an observer to expand the results of the present study. Last but not least, future studies could incorporate short video selfie cues that could reflect people’s personalities (e.g., acting) into machine algorithms to promote the efficiency and accuracy of machine learning in predicting personality traits based on the results.

## Conclusion

Short video selfies play an essential role in daily life. The study tested personality expression and perception in short video selfies through the lens model. Some particular short video selfie cues, such as acting, in revealing one’s personality traits were identified. Moreover, the results further indicate the relationship between the preference for short video selfies and demographic variables (i.e., gender and age). Theoretically, these findings expand the field of personality expression and perception on social networking sites and increase our understanding of personality expression and perception through the lens model. Practically, a few commercial applications could improve their recommender system by learning personality, thus promoting users’ experience.

## Data availability statement

The raw data supporting the conclusions of this article will be made available by the authors, without undue reservation.

## Ethics statement

The studies involving human participants were reviewed and approved by Ethical Committee of Philosophy School at Wuhan University. Written informed consent for participation was not required for this study in accordance with the national legislation and the institutional requirements.

## Author contributions

ZD: methodology, investigation, formal analysis, writing–original draft, and writing—review and editing. TX: conceptualization, investigation, formal analysis, and writing—review and editing. All authors contributed to the article and approved the submitted version.

## Funding

This study is part of a large-scale project entitled “Constructing the ‘circle of friends’ in global media from the perspective of intercultural communication,” funded by the MOE (Ministry of Education of China) Social Science Research Institute Grants (Project No. 22JJD860013).

## Conflict of interest

The authors declare that the research was conducted in the absence of any commercial or financial relationships that could be construed as a potential conflict of interest.

## Publisher’s note

All claims expressed in this article are solely those of the authors and do not necessarily represent those of their affiliated organizations, or those of the publisher, the editors and the reviewers. Any product that may be evaluated in this article, or claim that may be made by its manufacturer, is not guaranteed or endorsed by the publisher.

## Footnotes

^1^ www.sojump.com
